# Beyond words: analyzing non-verbal communication techniques in a medical communication skills course via synchronous online platform

**DOI:** 10.3389/fmed.2024.1375982

**Published:** 2024-04-18

**Authors:** Noor Akmal Shareela Ismail, Nanthini Mageswaran, Siti Mariam Bujang, Mohd Nasri Awang Besar

**Affiliations:** ^1^Department of Biochemistry, Faculty of Medicine, Universiti Kebangsaan Malaysia, Kuala Lumpur, Malaysia; ^2^Department of Medical Education, Faculty of Medicine, Universiti Kebangsaan Malaysia, Kuala Lumpur, Malaysia

**Keywords:** non-verbal communication, synchronous, feedback, online, medical students

## Abstract

**Background:**

Effective doctor-patient relationships hinge on robust communication skills, with non-verbal communication techniques (NVC) often overlooked, particularly in online synchronous interactions. This study delves into the exploration of NVC types during online feedback sessions for communication skill activities in a medical education module.

**Methods:**

A cohort of 100 first-year medical students and 10 lecturers at the Faculty of Medicine, Universiti Kebangsaan Malaysia (UKM), engaged in communication skills activities via Microsoft Teams. Sessions were recorded, and lecturer NVC, encompassing body position, facial expressions, voice intonation, body movements, eye contact, and paralinguistics, were meticulously observed. Following these sessions, students provided reflective writings highlighting their perceptions of the feedback, specifically focusing on observed NVC.

**Results:**

The study identified consistent non-verbal communication patterns during feedback sessions. Lecturers predominantly leaned forward and toward the camera, maintained direct eye contact, and exhibited dynamic voice intonation. They frequently engaged in tactile gestures and paused to formulate thoughts, often accompanied by filler sounds like “um” and “okay.” This consistency suggests proficient use of NVC in providing synchronous online feedback. Less observed NVC included body touching and certain paralinguistic cues like long sighs. Initial student apprehension, rooted in feelings of poor performance during activities, transformed positively upon observing the lecturer’s facial expressions and cheerful intonation. This transformation fostered an open reception of feedback, motivating students to address communication skill deficiencies. Additionally, students expressed a preference for comfortable learning environments to alleviate uncertainties during feedback reception. Potential contrivances in non-verbal communication (NVC) due to lecturer awareness of being recorded, a small sample size of 10 lecturers limiting generalizability, a focus solely on preclinical lecturers, and the need for future research to address these constraints and explore diverse educational contexts.

**Conclusion:**

Medical schools globally should prioritize integrating NVC training into their curricula to equip students with essential communication skills for diverse healthcare settings. The study’s findings serve as a valuable reference for lecturers, emphasizing the importance of employing effective NVC during online feedback sessions. This is crucial as NVC, though occurring online synchronously, remains pivotal in conveying nuanced information. Additionally, educators require ongoing professional development to enhance proficiency in utilizing NVC techniques in virtual learning environments. Potential research directions stemming from the study’s findings include longitudinal investigations into the evolution of NVC patterns, comparative analyses across disciplines, cross-cultural examinations, interventions to improve NVC skills, exploration of technology’s role in NVC enhancement, qualitative studies on student perceptions, and interdisciplinary collaborations to deepen understanding of NVC in virtual learning environments.

## 1 Introduction

Communication skills are important in building personal and professional relationships. In medicine, it is the basis for the relationship between a medical doctor and a patient. There are two types of communication which are, verbal communication and non-verbal communication. Non-verbal communication (NVC) is an important aspect of communication in conveying messages and can emphasize verbal communication. Compared to verbal communication, the area of non-verbal communication has been poorly explored.

Non-verbal communication encompasses the conveyance of information through bodily signals, encompassing elements such as eye contact, facial expressions, gestures, and vocal cues (paralinguistics) (1). An instance of this could be the act of smiling upon meeting someone, which signifies traits such as friendliness, acceptance, and openness. The utilization of non-verbal communication is ubiquitous, operating in myriad contexts, often occurring unconsciously. Therefore, it is important to study non-verbal means of communication based on the observation and analysis of physical movements compared to verbal communication, or the use of language to transfer information through written text, spoken or sign language. Recent studies show that communication skills among medical students are lacking, especially in areas of NVC and empathy toward patients (2–4). Feedback can improve the NVC of medical students during the communication process, especially facial expressions, body movements, body posture, silent intervals, and laughter (3). This technique is more meaningful and effective than verbal communication because it conveys a more dominant message to an individual after receiving feedback (3). In the event of a conflict, the message conveyed through NVC will be stored in the memory longer than verbal messages (5). The significance of NVC in conflict resolution lies in its ability to convey emotions, intentions, and attitudes non-verbally, thereby influencing the dynamics of interpersonal interactions. In conflict situations, NVC cues such as facial expressions, gestures, and body language can play a crucial role in either escalating or de-escalating tensions, fostering empathy, understanding, and collaboration among conflicting parties (2). By recognizing and interpreting NVC signals, individuals can better navigate conflicts, facilitate effective communication, and work toward mutually beneficial resolutions (6).

Online observation of NVC poses several limitations compared to face-to-face interactions. The digital medium may distort or diminish certain non-verbal cues, making accurate interpretation challenging (2). Factors such as poor video quality, lagging audio, or limited camera angles may obscure important NVC signals, leading to misinterpretations or incomplete assessments (7). Online observation lacks the immediacy and intimacy of in-person interactions, potentially hindering rapport-building and emotional connection between participants (1). Moreover, the absence of physical proximity in virtual settings may limit the range and subtlety of non-verbal behaviors that can be observed and analyzed (7). Subsequently, online observation may be subject to biases inherent in the digital platform, such as selection bias in participant recruitment or observer bias in data interpretation, further complicating the validity and reliability of NVC research conducted online (8).

Therefore, this feedback process needs to be given special attention because it is often neglected in studies related to effective communication. Communication skill activities will be more appreciated if a person’s behavior can be observed more closely in non-verbal terms (6). The era of COVID-19 has transformed learning communication skills online. This impacts the detection of non-verbal communication that can be observed online and how it affects the effectiveness of communication with patients. This is because when online teaching is conducted, the observation of NVC through synchronous online applications is limited, so it can cause misunderstandings and thus can result in less effective communication. Therefore, NVC should be given extra attention especially when communication is done online synchronously so that the information conveyed is clear and fulfils its purpose. The observation of non-verbal communication (NVC) is limited to the visual cues captured by camera imagery, typically focusing on the upper body, in contrast to the comprehensive interaction afforded by face-to-face encounters (6, 8, 9). The scarcity of research dedicated to NVC diminishes its recognition as a pivotal component of effective communication. Therefore, this study aims to explore the types of NVC by lecturers during feedback sessions through a synchronous online application.

## 2 Materials and methods

### 2.1 Study design

Qualitative methods with an observational research approach were used to explore the types of non-verbal communication (NVC) among lecturers in giving feedback and gaining acceptance of Year 1 medical students in getting feedback on the performance of their communication skills in the Professional and Personal Development module. This feedback was given by the lecturer to improve student’s performance. This observational study is important to see the types of NVC that are given online synchronously. The feedback process was recorded through the Microsoft Teams application for student reference so that students can make self-reflection and use all the feedback given to improve their weaknesses during communication skill activities.

#### 2.1.1 Observational studies

An observational study was chosen as a research method to see the types of feedback given by lecturers on student performance. It only required one researcher to view the video footage and this method is a thorough method of collecting, recording, and analyzing data. Through this study, deductive and inductive thematic data analysis (10) was used to answer the questions and objectives of this study. Observational research also provides a platform to add new criteria or types of NVC based on observations from one individual lecturer to another. All aspects of the researched material were explained through data analysis from the feedback recording.

#### 2.1.2 Reflective writing

Reflective writing has become one of the components of continuous assessment in medical education. It is a practice to look back at a student’s performance and has become the core of the concept of learning from experience (11). This method is practiced to improve a student’s ability to develop critical thinking, analytics, and cognition (12). Self-reflection is important to see if any new perspective was learned and helps improve student progress in learning a skill. Reflective writing is an additional research tool that helps to explore the effect of feedback from lecturers and the acceptance of the feedback from the student’s perspective and how it can be used to improve their performance in communication.

### 2.2 Validity and reliability

There are two challenges in observational studies that researchers need to see, namely validity and reliability. The selected sample must have characteristics of the population that is being studied so that the study can give a general statement/picture about a population (13). Observational studies have low validity, meaning that the findings cannot be generalized to other populations (14). It is also because there is no control group comparison. However, the reliability of this study was high because the study observer was the same person all data or input obtained from the small group discussion activity session was processed by the researcher and no case of bias or observer variation occurred. This study was conducted well as all respondents (students and lecturers) gave their full cooperation, instructions given to the participants were clear and the format of the study was systematic.

In this study, maintaining methodological consistency was paramount. Before data collection commenced, a team of three researchers convened to establish standardized observation protocols, guided by a predetermined checklist. To acquaint themselves with the protocol, a trial feedback session was conducted, serving as both a training and standardization exercise. A standardized evaluation method was then selected to ensure adherence to predefined non-verbal communication (NVC) criteria and maintain consistency across observations. Furthermore, the standard-setting process involved three independent researchers, who collectively established benchmarks for specific NVC types.

The observation process consisted of three rounds. Initially, individual marks were assigned during the first round of observation. Subsequently, collaborative discussions ensued during the second round to refine observations and address any discrepancies. Finally, a third round was conducted to collectively discuss and reach a consensus on the evaluation of each NVC parameter. To facilitate the inductive thematic analysis, specific keywords were established to describe different NVC types, streamlining the analysis process.

### 2.3 Study location

This study observed the types of non-verbal communication of lecturers on the performance of Year 1 medical students who undergo communication skill activities in the Professional and Personal Development module which is conducted online synchronously. The course content encompassed the demonstration of multiple soft skills and aspects of professionalism pertinent to university settings. These included proficiency in self-management and coping strategies, fostering a caring attitude and sensitivity toward both personal needs and those of significant others, adeptness in critical thinking and interpersonal communication for learning and everyday life scenarios, advocacy for healthy lifestyles while recognizing the implications of unhealthy behaviors, particularly within the context of medical student life, and extending these principles to the broader public. Additionally, the course also emphasized the cultivation of a team-oriented spirit of collaboration across diverse professions, underpinned by principles of integrity and passion. This study was conducted through the Microsoft Teams online application (15). All student activities and lecturer feedback were recorded for data analysis purposes.

### 2.4 Population of study and sampling

The population of this study consisted of Year 1 undergraduate medical students of UKM who are undergoing the Professional and Personal Development module (FFFF1813) in the 1st semester of the 2020/2021 session. This group of first-year students have never undergone any formal feedback session neither the Professional and Self-Development module. Purposive sampling was used and the participants for this study were selected according to the criteria set by the researcher. The inclusion criteria for this study were as follows: 1. First-year undergraduate medical students who have never undergone the Professional and Personal Development module; 2. Students who give written consent to participate in this study. The exclusion criteria were first-year undergraduate medical students who have undergone the Professional and Personal Development module (repeat students) and students from years other than the first year. Students who agreed to be participants were divided into groups of 10 people per group according to the session that had been set by the researchers. All communication skill activities and feedback sessions were recorded through the Microsoft Teams application. This activity was conducted for 2 h for each group. Then, a video recording of the feedback was given to each student. After the communication skill activity and feedback session from the lecturer, students wrote a reflection on the activity and how the feedback from the lecturer could be put into practice to improve their weaknesses during the activity.

### 2.5 Sample size

**Figure 1:**
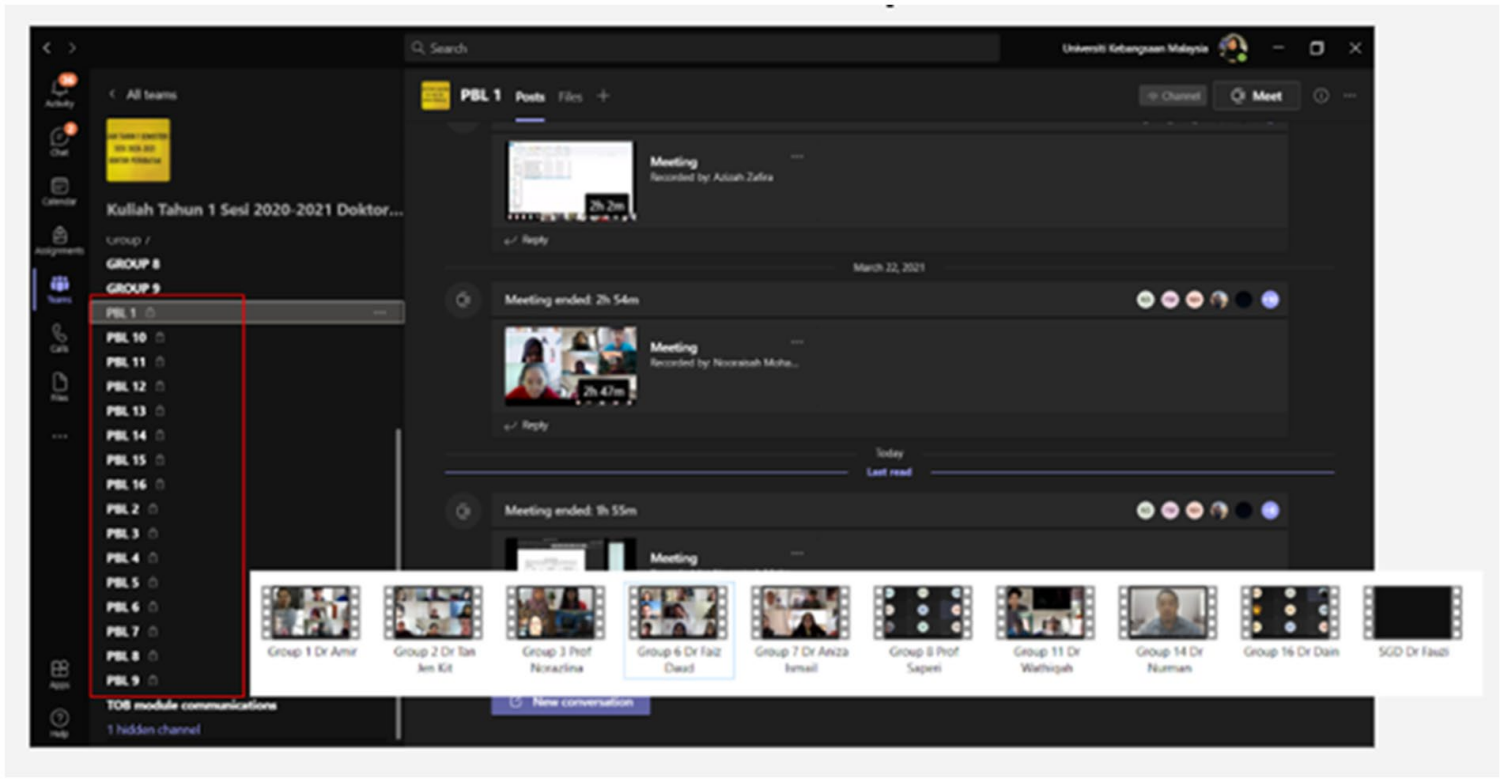
Communication skill activity conducted online synchronously and recorded via Microsoft Teams application.

Researchers set 10 participants for each group and a total of 10 groups (*n* = 100) participated in this study. This is to facilitate the recording process that took place in a group and was recorded through the application in Microsoft Teams (Figure 1). Sample adequacy in qualitative inquiry refers to the evaluation of whether the sample’s composition and size are suitable and relevant for the research objectives. This assessment plays a crucial role in determining the quality and trustworthiness of qualitative research. It involves ensuring that the chosen sample adequately represents the population of interest, possesses diversity where applicable, and is of a size sufficient to yield meaningful insights and achieve saturation, thereby enhancing the credibility and validity of the study findings (16). Since this was an observational study, the study sample did not need to be large, and the researcher had set observations in 10 different groups to reach conclusions for this study. For reflective writing, only two groups of study participants (*n* = 20) were required to do reflective writing about their performance through communication skill activities and acceptance of feedback given by their lecturer.

### 2.6 Data collection and analysis

**Figure 2:**
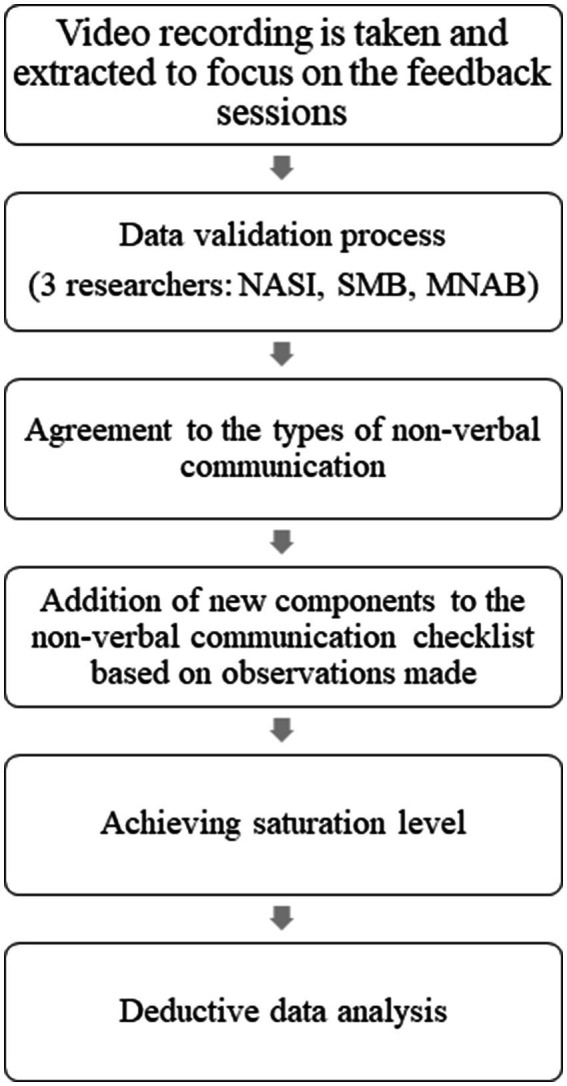
Deductive data analysis process in the observation of non- verbal communication via video recording.

Before the commencement of recordings, all participants received a comprehensive briefing. They were informed that the recordings would be utilized exclusively for research purposes and would be accessible solely to the researchers involved in the study (NASI, SMB, MNAB). Data taken during small group discussion activity was recorded using the Microsoft Teams online application. Then the video recording was viewed again. A checklist of types of non-verbal communication, such as body position, facial expressions, voice intonation, movement, eye contact and paralinguistics was created based on the observation of video recordings and literature studies (Supplementary Table 1). The process of verifying the types of non-verbal communication was done with two independent researchers who were not involved in the data collection process. This process was carried out by looking at 2 different types of videos. Three researchers (NI, SB, MB) marked the types of non-verbal communication and the time the behavior was observed. Then, these three researchers (NI, SB, MB) presented their observations and discussed the types of non-verbal communication observed. Next, NI, SB, and MB continued to observe the types of non-verbal communication until it reached a saturation level. This process is known as deductive thematic analysis (Figure 2) which was presented in a checklist (Supplementary Table 1).

**Figure 3:**
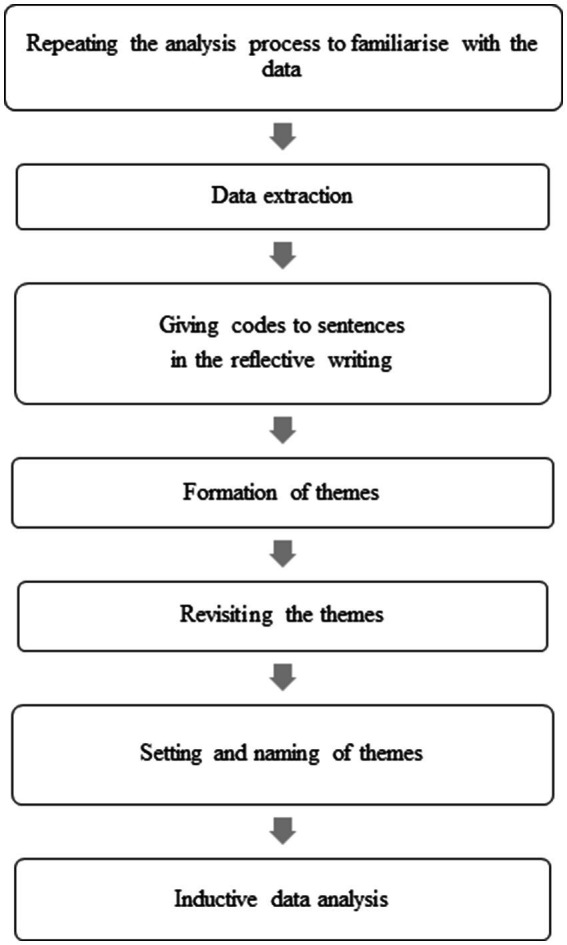
Inductive data analysis process of student acceptance of non-verbal communication of lectures through reflective writing.

**Figure 4:**
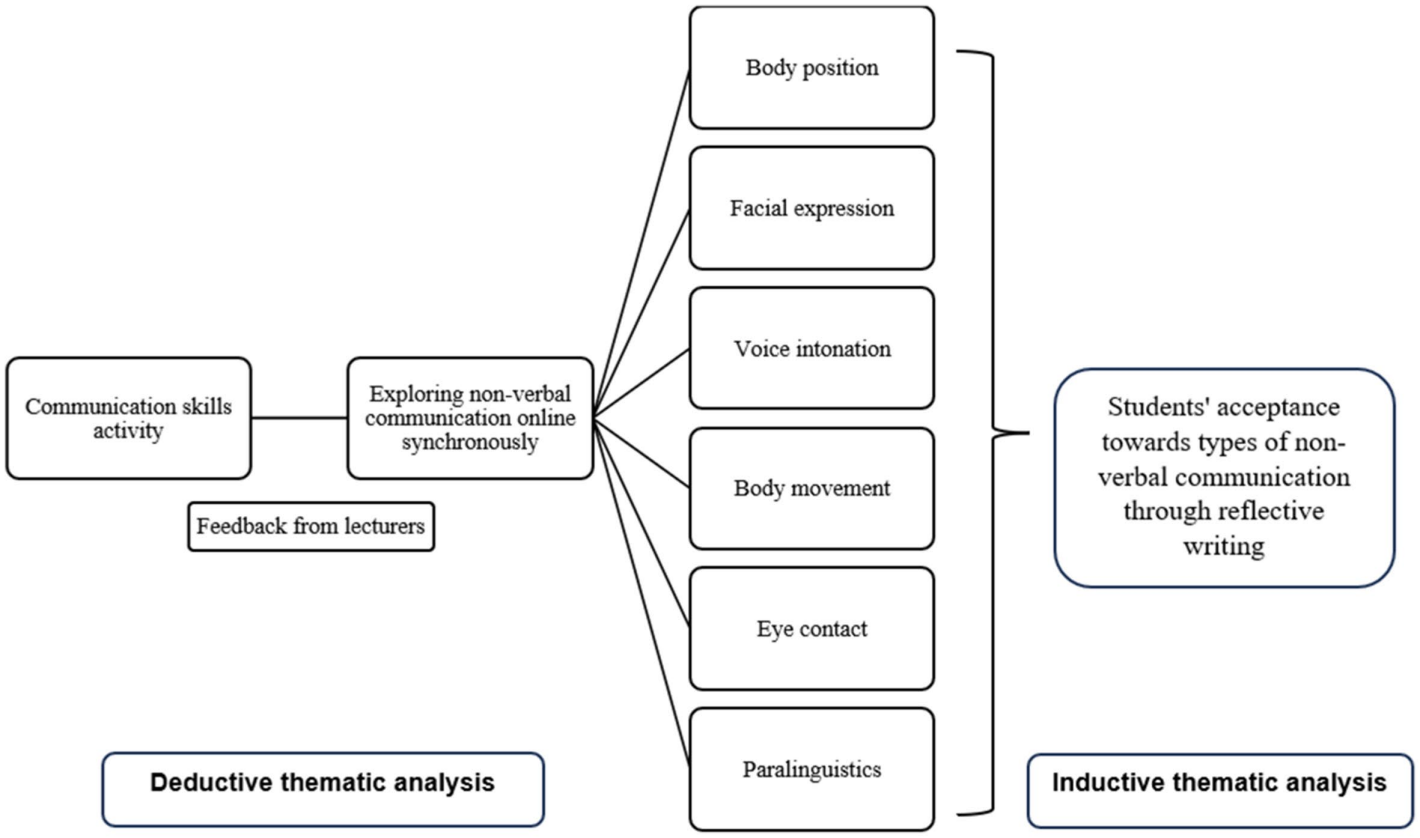
Flow chart of the data collection process.

For the analysis of the reflective writing, students were given the task of writing a reflection on their performance and the feedback given by the lecturer after the small group discussion. Reflective writings were collected according to the number of samples set (*n* = 20). A purposive sampling technique was used from two groups consisting of a young lecturer and a professor-ranked lecturer. Before the final analysis was obtained, the researcher marked similar themes and separated them according to main themes, sub-themes and statements involved (Supplementary Table 2). This process is known as inductive thematic analysis (Figure 3). This involves the process of repeating data, extracting data, assigning codes to sentences, forming themes, revisiting themes, determining and naming themes, and analyzing the study (17). Figure 4 shows the entire data collection process of this study. Confidentiality was maintained where only the researcher and the research team could access the information provided by study participants. Any name or personal identification was not used in this study and the information provided by the participants was recorded as K1P1 to refer to group 1 and the first participant, and so on.

## 3 Results

### 3.1 Types of non-verbal communication

Non-verbal communication encompasses the deliberate or involuntary behaviors exhibited by individuals during interpersonal interactions. In this study, feedback sessions were conducted within a tranquil environment, specifically designated personal rooms equipped with computers featuring cameras and microphones. This controlled setting aimed to minimize potential disruptions, such as interference from familial influences, thereby optimizing the focus and concentration of the subjects involved in the study. NVC can be seen through a person’s body movements, eye contact and voice intonation. In this study, six types of NVC could be observed online, namely body position, facial expressions, voice intonation, movement, eye gaze and paralinguistics. For these types of NVC, a total of 10 lecturers were observed.

#### 3.1.1 Body position

Body position only can be observed from chest level up to the head when captured online. Therefore, the observation is limited to the way the body is positioned toward the camera. Table 1a shows the percentage of lecturers who show the most dominant body position. This includes body leaning forward (100%), sitting upright (90%), bending over (0%), body position to the left (10%) and right. This shows that the most dominant body position exhibited by all lecturers was leaning forward and toward the camera. This shows that all the lecturers were very interested and worked hard to give feedback to the students. Throughout the feedback session, all the lecturers were seen sitting upright except for one lecturer who seemed to be sitting sideways to the left. This shows that the lecturers were always focused on delivering information to students.

**Table 1:** Types of non-verbal communication observed during the feedback session.

Types of non-verbal communication observed	Percentage (%)
a. Body position	
Leaning forwards	100
Sitting upright	90
Bending over	0
Body to the left side	10
Body to the right side	0
b. Facial expression	
Looking directly into the camera	100
Smiling	100
Laughing	30
Frowning	10
Raising eyebrows	20
c. Voice intonation	
Dynamic and cheerful voice	100
Monotonous	0
Raising voice	0
Unclear voice	0
Emphasizing words	10
d. Movements	
Hands touching face	50
Hands touching chin	50
Hands touching nose	50
Hands at the mouth	50
Nodding the head	20
Shaking the head	20
Body swaying to the left/right	10
Body swaying to the front/back	10
Free movement of hand	20
Hands on the hair	20
Scratching the body	10
Scratching the forehead	10
Squinting eyes	10
Rubbing eyes	10
Touching shoulders	10
Touching frame of glasses	50
Clapping hands	20
Scratching behind the ears	20
Signalling OK	10
e. Eye contact	
Looking to the right	60
Looking to the left	80
Looking up	30
Looking down	70
f. Paralinguistics	
Word fillers (*urmm/”okay”/ lah/ “so”/”that’s it”*)	100
Sound of being shocked	0
Long breath of relieve	0
Pause for a moment	100
Clearing throat	10
Speaking in a fast pace	0
Speaking in a slow pace	0

#### 3.1.2 Facial expressions

The lecturer’s facial expressions are very important in conveying information and feedback to students. This is because the lecturer’s facial expressions can be seen directly through the camera. Table 1b shows the description of the lecturer’s facial expressions that were observed through the online application. These include looking directly into the camera (100%), smiling when speaking (100%), laughing (30%), frowning (10%), and raising eyebrows (20%). The most dominant facial expression was looking directly at the camera when giving feedback. This is important to show the lecturer’s sensitivity and focus in delivering information. Some lecturers show cheerfulness by inserting jokes interspersed with laughter and smiles. This was to create a cheerful and comfortable atmosphere for students to receive feedback. However, some lecturers frowned and raised their eyebrows as the feedback session progressed. This shows a serious tone in conveying information.

#### 3.1.3 Voice intonation

In addition to facial expressions, voice intonation also plays an important role in interaction with students. Cheerful voice intonation is essential in evoking a comfortable atmosphere in learning especially if it is done online. The observed types of voice intonation were monotonous, raising the voice, unclear and giving emphasis to a word (Table 1c). Through virtual observation, all lecturers had a dynamic and cheerful voice intonation. None had a monotonous or raised voice and was unclear throughout the conversation. There was a lecturer who always emphasized words. This was intended to attract students’ attention to the information presented.

#### 3.1.4 Movement

There were limitations in identifying the types of movements that can be recorded through the online application, including those related to touching the face, hand movements, and even the head. Table 1d shows the types of movements that were observed. The most dominant movements (50%) were hands touching the face, chin and nose and touching the glasses frame when giving feedback. This is followed by nodding, shaking the head, touching the hair, making free-hand movements, clapping and scratching behind the ears (20%). The least type of movement observed was body movement to the left/right / front/back. This also includes scratching the body, scratching the forehead, squinting, rubbing the eyes, holding the shoulder, and giving the OK! sign (10%).

#### 3.1.5 Eye contact

Eye contact is one of the types of non-verbal communication that is often observed in everyday communication. However, in online communication, this type of NVC is very meaningful, especially in conveying information. Therefore, lecturers must look toward the camera or computer screen when speaking. Overall, the most dominant eye contact was when 80% of lecturers looked to the left when giving feedback. This is followed by looking down (70%), to the right (60%) and up (30%; Table 1e).

#### 3.1.6 Paralinguistics

Paralinguistics include types of non-verbal communication that involve sounds. This includes saying “aaah,” “okay,” “laaaah,” “so,” “hmmm..,” surprised sounds, sighing sounds when complaining and clearing throat. Table 1f shows observations on the types of paralinguistics exhibited by lecturers. All lecturers seem to always pause to think of the best words in giving feedback to students (100%). This situation was filled with sounds like …urmm/ “okay”/ lah/ “so”/“that’s it” (100%). There was a lecturer who always cleared his throat when he paused for a moment (10%) and there was no observation of the sound of sighing or being surprised. The lecturers in this study were also not seen to speak too fast or too slowly (0%).

### 3.2 Student acceptance of non-verbal communication

**Figure 5:**
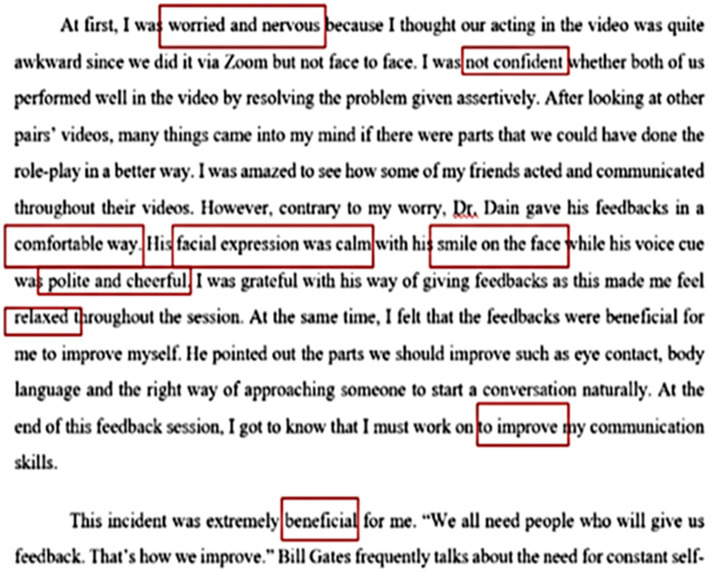
Examples of student’ reflective writing and Keyword detection for theme formation using the thematic analysis technique.

After completing the communication skill activity, students were required to write a reflective essay expressing their feelings toward the lecturer’s non-verbal communication. A total of two groups were selected in this study (*n* = 20). Each student’s writing was extracted to form the same theme (Supplementary Table 2). An example of reflective writing is in Figure 5. The formation of subthemes from the same keywords was used to form the main theme. The findings of this study found 4 types of main themes, namely students’ feelings before receiving feedback, while receiving feedback, after receiving feedback and the type of non-verbal communication that was observed. This write-up will be presented according to sub-themes and selected statements related to the keywords that make up the sub-themes and then the main theme.

### 3.3 Theme 1: students feelings before receiving feedback

At the beginning of the reflective writing paragraph, most students recorded their feelings before receiving feedback. This includes feelings of nervousness, restlessness, fear and apprehension.

#### 3.3.1 Nervousness

The feeling of nervousness in the context of this study can be defined as a feeling of unease. Students have stated that they felt nervous before receiving feedback from the lecturer. This is because they were not confident in their performance in the communication skill activities. This was seen through examples of writing below:

*“I felt very nervous and worried about the feedback of the facilitator and my other friends on my task”* (K2P1).

*“I was worried and nervous because I thought our acting in the video was quite awkward since we did it* via *Zoom but not face to face”* (K2P3).

#### 3.3.2 Restlessness

Feeling restless is different from feeling nervous. Students wrote down their feelings of anxiety before the feedback session. This is because they had palpitations during the face-to-face feedback session, even if it was conducted online. Examples of students’ writing were as follows:

*“I was already feeling anxious about what would be the feedback towards the outcome”* (K1P5).

*“I felt really anxious and scared of what my friends and my facilitators would comment on my performance”* (K1P6).

*“I was anxious and curious about how my teammates and our facilitator’s opinions on the task”* (K1P3).

*“I was worried and nervous for this session because I was scared that I would be the worst student for this task.”* (K1P1).

#### 3.3.3 Fear

Fear was also expressed by students in this reflective writing. The term fear can be defined as someone who feels worried (doubt, apprehensive), and is often associated with a feeling of anxiety. It can be seen in the student’s writing below:

*“I was worried and nervous for this session because I was scared that I would be the worst student for this task.”* (K1P1).

*“I was kind of scared as this was our first roleplay video”* (K2P2).

*“We all still feel anxious, scared, and worried when receiving feedback as we are worried about how the facilitator looks at us and perceives us”* (K2P4).

#### 3.3.4 Apprehension

In addition to fear, students also felt apprehensive before receiving feedback from their lecturer. This is because they were worried about their performance in the communication skill activities. The word apprehensive is interpreted as when a person is somewhat anxious or afraid of something happening, feels restless or uneasy (because of thinking about something), and worries about something. This can be seen in the statements below:

*“I was not confident whether my performance was good enough to be accepted.”* (K1P1).

*“I felt a little bad about myself as I could not give a proper presentation”* (K1P2).

*“Many things were running through my head thinking if there were parts that I could have done in a better way, after looking at some of my peer’s performance”* (K1P9).

*“I think I’m not good at communication and need some improvement”* (K1P10).

*“I thought our acting in the video was quite awkward since we did it* via *Zoom but not face to face.”* (K2P3).

### 3.4 Theme 2: students feelings when receiving feedback

Because students felt nervous before receiving feedback, they felt relieved after receiving feedback. They also feel very comfortable and happy with the way the lecturer gives feedback.

#### 3.4.1 Relief

Relief can be described as peace of mind. Although students felt nervous and anxious before listening to feedback from their lecturer, they could breathe a sigh of relief when listening to the feedback from the lecturer and this has been written by several students. This is because the lecturer has shown calm facial expressions and dynamic voice intonation to create a comfortable atmosphere. Examples of their writing are as follows:

*“That kind of eases us out of tension towards our feedback.”* (K1P5).

*“I would feel a slight relief whenever he/she puts on a smile on their face, though I must admit I would be a little nervous if they had a stern face”* (K1P8).

#### 3.4.2 Comfortable

Students felt comfortable with the entire feedback session that was conducted. The feeling of comfort can be interpreted as feeling good and pleasant in a situation. This has to do with the cheerful tone and facial expression of the lecturer. This feeling can be described as below:

*“His voice cue was not in an angry tone throughout the session. This made us feel more focused and excited throughout the session.”* (K2P8).

*“I was grateful with his way of giving feedback as this made me feel relaxed throughout the session.”* (K2P3).

*“I even felt very calm as looking Prof. smiled to us and tried to explain our mistakes.”* (K1P10).

*“This brought a great atmosphere for all of us during the session the other day.”* (K1P9).

*“I have never felt uneasy or disheartened by how my facilitators presented their feedback”* (K1P8).

*“She definitely sets the mood right.”* (K1P6).

#### 3.4.3 Happy

Happiness is a feeling of great joy and magnanimity toward a situation that a person feels. In a comfortable atmosphere created by the lecturer during the feedback session, students stated that they felt happy with the feedback that has been given as in the statements below.

*“I am very happy and grateful to learn the ways of communication skills with my facilitator (lecturer)”* (K2P1).

*“This really made me relieved and happy”* (K1P4).

*“My group members and I feel very happy because we receive a lot of positive feedback from our friends and facilitator”* (K2P2).

### 3.5 Theme 3: students feelings after receiving feedback

After the feedback session, students made a self-reflection on the lecturer and their performance. This included appreciation for the lecturer, understanding of the feedback that had been given, determination to improve one’s performance, making improvements and further self-reflection.

#### 3.5.1 Appreciation

The term appreciation in the context of this study can be expressed as a description or act of gratitude and respect to someone. Students appreciated the feedback given by the lecturer. This can be seen through the statements below.

*“I felt the feedback session was very meaningful and great”* (K1P2).

*“I believe that it has helped me a lot and I still think the same way as I am right now”* (K1P3).

*“Prof guided me towards the right path in enhancing my communication skills.”* (K1P9).

*“I am very happy and grateful to learn the ways of communication skills with my facilitator”* (K2P1).

*“He pointed out the parts we should improve such as eye contact, body language and the right way of approaching someone to start a conversation naturally.”* (K2P2).

#### 3.5.2 Perception

Perception is the process of creating an image in the heart or mind about something. The feedback session in the communication skill activity also improved the student’s perception of the importance of communicating well. This can be seen through examples of student statements as below:

*“Open-minded towards the acceptance of criticizing comments and viewing them from a humble angle”* (K1P7).

*“It is very important to communicate with eye contact and the appropriate speed which can make us deliver our message more clearly and easily during the conservation”* (K2P1).

*“We also learnt that we must adjust to different situations and audiences, especially later on when we start working as a doctor”* (K2P2).

#### 3.5.3 Determination

Determination can be defined as a will (desire, ambition) that is very strong. In the context of this reflective writing, the students determined to improve their communication skills. After receiving feedback from the lecturer, the students felt they were determined to improve themselves, especially in communication. This is because students received good feedback from lecturers who showed different types of non-verbal communication that provided a non-threatening and comfortable space for students. Therefore, they were able to receive such feedback and improve existing deficiencies. This can be seen through examples of writing below:

*“To know my weaknesses and strengths in communication skills to be more confident in improving my personal skills”* (K1P5).

*“I plan to continue improving my communication skills”* (K1P8).

*“I have learned a lot regarding communication skills and how to implement it in my daily life”* (K2P10).

#### 3.5.4 Improvement

The definition of improvement can be said to be a process that involves the re-evaluation of something. Improvement in the context of this study refers to communication skills. It can be explained through student statements as below.

*“I plan to continue improving my communication skills”* (K1P8).

*“I am sure now that this is something I am working on to be better”* (K1P9).

*“I also believe that it is very important to accept ourselves and learn from mistakes because avoid making the same mistake again in the future and continue to grow stronger as we through our life.”* (K1P10).

#### 3.5.5 Self-reflection

The nature of self-reflection after receiving feedback is important to build self-motivation in improving existing skills. Reflection is defined as the act of analyzing oneself for self-change. Students felt they were more open to receiving criticism or comments if the lecturer set a comfortable atmosphere. This could be explained through reflective writing excerpts as below:

*“I know where I’m lacking and how I should fix it”* (K1P7).

*“I plan to continue on improving my communication skills”* (K1P8).

*“I am sure now that this is something I am working on to be better.”* (K1P9).

*“I also believe that it is very important to accept ourselves and learn from mistakes because avoid making the same mistake again in the future and continue to grow stronger as we through our life.”* (K1P10).

*“I am no longer to be afraid and nervous when receive feedback from someone”* (K2P1).

*“I would not assume I have fully mastered this communication skill and I would like to talk to more people”* (K2P10).

### 3.6 Theme 4: observation of lecturer’s types of non-verbal communication

Through students’ reflective writing, four main themes could be extracted, which were the types of non-verbal communication (NVC) of lecturers observed by students. Among the most dominant NVCs told through students’ reflective writing were facial expressions, voice intonation, eye contact and smile.

#### 3.6.1 Facial expressions

A person’s facial expression is the most observed during communication, especially through online applications. This is because the camera only focuses on a person’s face and when speaking, facial expressions need to be refined to get a message that is parallel to the verbal message. Therefore, based on the students’ reflective writing, it was found that the lecturer’s facial expressions played a very important role in making the feedback process work effectively. This is shown in the excerpts below:

*“I felt comfortable as our facilitator did not show any expression of displeasure on her face.” (K1P2).*

*“Prof’s facial expression was very calming, and I could feel the positive vibes from Prof”* (K1P1).

*“He always puts on a smiling face and passes his comments in a very polite manner as to not hurt our feelings”* (K2P3).

However, some students stated that sometimes the lecturer gave serious facial expressions, to emphasize the message of feedback given.

*“My facilitator uses a more neutral and serious expression when giving comments to make sure that I managed to get the main message and not repeat the same mistakes in the future”* (K1P4).

There was a student who stated that the lecturer did not show any positive facial expressions and made them wonder about the message conveyed.

*“There are times where the facilitator would just give us a blank facial expression, and at those times I would feel rather confused or I tend to overthink and feel nervous for his/her feedback”* (K1P8).

#### 3.6.2 Voice intonation

Voice intonation is a state of fluctuation or height of voice tone when speaking. In addition to facial expressions, voice intonation also plays an important role in providing a calm atmosphere for a feedback session to take place. This is because, based on the students’ reflective writing, they felt very anxious and nervous before receiving any feedback.

*“…tone from the facilitator were really positive and full of motivation that makes me feel comfortable and able to receive the advice effectively.”* (K1P1).

*“She delivers her comments in a calm facial expression and a gentle tone.”* (K1P5).

*“Soft spoken and encouraging towards our efforts and capabilities”* (K1P8).

There were also comments stating that a firm and serious voice tone helps ensure that the conveyed message is accurate and tells students to take it seriously when it comes to improving their performance in communication.

*“My facilitators use a serious tone when giving feedback to make sure that I got the take-home message.”* (K2P8).

#### 3.6.3 Eye contact

Eye contact is the movement of the eyes to the right or to the left without moving the head. Since the entire feedback session was face-to-face and the lecturer looked toward the camera, eye contact could also influence the message conveyed. It can be seen through student writing as below:

*“One thing would be that making eye contact when appropriate is important when talking to someone to ensure that the conversation is bidirectional and for both to receive inputs from it.”* (K1P7).

#### 3.6.4 Smile

A smile can be described as the movement of the lips which is charming and can soften the heart. A smile can affect the students’ feelings when receiving feedback from the lecturer. This is shown in the excerpt below:

*“Prof put on a pleasant smile, and I did not feel any sense of discomfort from her.”* (K1P5).

The findings of this study have been organized according to the themes that have been processed through deductive analysis for the types of non-verbal communication and also inductive analysis for students’ reflective writing. Through the findings of this study, it can be concluded that several types of non-verbal communication can be observed online. Students’ acceptance when receiving feedback is good if the lecturer’s non-verbal communication supports the information conveyed by providing a cheerful and comfortable atmosphere.

## 4 Discussion

### 4.1 Observation of types of non-verbal communication online

Non-verbal communication (NVC) is the transfer of information using body language including eye contact, facial expressions, gestures, and voice (9). NVC is a habit and often reinforces the message to be conveyed through oral communication. Therefore, it is important to study non-verbal means of communication-based on observation and analysis of physical movements, using language to transfer information through written texts, spoken or sign language. Recent studies show that communication skills among medical students are lacking, especially in the context of NVC and empathy toward patients (2–4). Feedback can improve medical students’ NVC during communication, especially through facial expressions, movements, body posture, silent intervals, and laughter (8). Therefore, this study must be conducted by observing how a feedback process is delivered especially through a synchronous online platform.

#### 4.1.1 Body position

Body position is important to complement the way feedback is given. This is easily captured by the camera as it can detect the body’s position toward the camera. In the observation made online synchronously, the position of the body can be seen mainly from the chest level to the head. The overall position of the body can be seen well, whether it appears to be sitting upright or leaning to the left or right. A good position for video recording is sitting upright and facing the camera. This shows that lecturers are always alert and ready to give feedback (18). Under this theme, most lecturers were seen sitting straight and upright (90%) and leaning toward the camera (100%). However, one lecturer was seen to always lean more to the left, which indicates a comfortable position when giving feedback (19).

#### 4.1.2 Facial expressions

Facial expressions play an important role in understanding a person’s feelings as an explanation of what the words are trying to convey. Various atmospheres, emotions, and attitudes can affect the learning environment positively or negatively. Therefore, these factors can be presented through various expressions (20). The human face can be so expressive that it conveys countless emotions without having to say a word. Facial expressions of happiness, sadness, anger, surprise, fear, and ugliness are similar across all cultures and societies around the world (21). Observations through this study show that all lecturers (100%) were seen smiling when giving feedback which gives a sense of safety and comfort to the students. Smiles are necessary to increase the sense of satisfaction with the activities that have been done by students (22, 23). Smiles provide positive feedback and involve the affective domain by conveying comfort, trust, friendliness, interest, joy, or surprise (24). A frown conveys displeasure, disapproval, and anger, a deadpan expression conveys disbelief, low energy, and disinterest (25). However, none of the lecturers frowned during the feedback session. Conversely, a lack of facial expression can be boring and should also be avoided (26). Therefore, facial expressions should always give a positive message to make students feel comfortable in receiving feedback.

#### 4.1.3 Voice intonation

A cheerful voice intonation can attract the attention of students to focus fully on feedback (27, 28). However, a cheerful and dynamic voice will complement the movement of the body, through appropriate tone and volume, to ensure the correct message is delivered (24). It can be seen from this study that all the lecturers delivered their feedback with a dynamic tone of voice to create a comfortable and safe zone for students to receive feedback. It can be measured through online platforms (5) and via student reception to voice intonation. Intonation can be associated with feelings of joy, anger, and sadness (29). A study on feedback from lecturers through audio recordings showed students can well receive the feedback given if the intonation of the lecturer’s voice is cheerful and not frightening (30). Therefore, a cheerful and calm voice intonation is essential in delivering the message to the student that the feedback session should not be avoided and should take place in a comfortable environment. The emphasis on the message delivered can also be reinforced with appropriate vocal intonation.

#### 4.1.4 Body movement

Body movement is the essence of a thousand and one messages that can be conveyed through NVC. For example, you may be able to gesture or use your hands when debating or speaking passionately as an example of NVC to show that you are a very focused and serious person when speaking. It also gives an impression to someone that your words or messages can be trusted. However, some body language can have very different meanings between cultures around the world. From 10 observations of body gestures observed through a synchronous online platform, large hand movements were seen to be the most dominant of all body gestures (100%). The most obvious indicator used to emphasize the information conveyed was hand movement within the camera frame. Observations in this study were in line with previous studies that showed lecturers extending their hands, with the palms facing slightly toward the camera (27). This shows a willingness to communicate and share ideas. It turns out that this gesture needs to be made to repeat the information given. However, if this gesture is done excessively, it may distract people to focus more on the hand than the idea or information conveyed. Movement of one’s hands and arms can also emphasize one thing; and continue to attract attention to determine whether something is important (31). This can be seen among lecturers who show good signs of asking for confirmation from students. Hand movements also show how enthusiastic a person is in conveying information (26). Small movements such as scratching the face and body and touching the glasses are normal, if not done regularly and deliberately.

#### 4.1.5 Eye contact

Since visual perception is dominant among most people, eye contact is a very important type of NVC. Information can be conveyed by just looking at someone, such as interest, affection, hostility, or attraction. Looking into someone’s eyes when talking is also important in ensuring that the conversation remains interesting and it can also tell whether a person is interested and reacts to the message conveyed (21). Therefore, if feedback is given through an online platform, the individual must look toward the camera as if looking into someone’s eyes. If feedback is given in a small group, looking at the participant’s screen will give the effect of looking without staring. Looking left and right when giving feedback is common as a process of remembering information (32). However, it should be avoided from being done excessively to prevent a reduction in student’s focus levels (21).

#### 4.1.6 Paralinguistics

Emphasizing conveyed messages through sound is a habit done by individuals. It is a habitual process in non-verbal communication techniques starting from children (33) up to adults (34). Paralinguistics is a sound that occurs with or replaces words, including tone of voice; and sounds of sighing, crying, and other non-verbal sounds (21). This can be reflected by the words “errrm,” “okay,” “aaaah” which are common in everyday conversation but should be reduced to avoid unnecessary disruption of the feedback process (35, 36). However, for every process of giving information in a conversation or speech, pausing for a moment is important to ensure that every message conveyed is easily understood (37). Therefore, an individual should balance the frequency of pausing for a moment, so that the information conveyed is not interrupted. Every activity that uses language or tests affective skills will involve paralinguistics. For example, through communication activities using a foreign language, paralinguistics is widely used especially in terms of voice quality and voice emphasis to explain the message conveyed (38). It turns out that the use of paralinguistics is important to help the delivery of information and it can influence how individuals receive it.

### 4.2 Reflective writing

Reflective writing is an effective way for students to integrate professional experience and academic learning (39). When used effectively, reflective writing can improve students’ understanding of the knowledge concept being studied. This approach can help students to critically evaluate the nature of their professionalism for lifelong learning (40). In this study, students made self-reflection on their performance in communication skills and how non-verbal communication by their lecturers conveys messages during feedback sessions.

#### 4.2.1 Student’s feelings before receiving feedback

Students’ acceptance of the feedback session from their lecturer increased the feeling of nervousness and anxiety among them. This is because students felt that their performance was not satisfactory when performing communication skill activities. The observations that have been carried out in this study were consistent with various other studies, especially through learning a new language course, where students feel that before feedback is given, they express feelings of nervousness and anxiety (41, 42). This is because feedback is meant to show their weaknesses for improvement (43). They were also worried that their performance did not meet the expectations set by the lecturer (44). This feeling was normal, especially among medical students who have high expectations of their performance (45). However, this feeling can be reduced if feedback is given via video recording, rather than synchronously online (7, 46–48) and then students would be able to make better self-reflection and improvement (8, 38). Students who have high feelings of nervousness, anxiety, fear and apprehension before receiving feedback can be associated with having a good learning curve and a high self-reflection process (49). Although this kind of feeling is unavoidable, it may be reduced by providing a comfortable learning environment especially when an activity takes place and when receiving feedback from the lecturer.

#### 4.2.2 Student’s feelings when receiving feedback

The findings of this study show that students felt very relieved, comfortable, and happy to receive feedback given by the lecturer. The same observation was also recorded in other studies that show that the role of lecturers is to convince students of their performance and strive to improve without feeling sad and afraid (50, 51). Students also need to be often reminded that the feedback process is important as an intermediary medium in delivering accurate information for improvement along with self-reflection (38, 52). This is also related to the negative feelings and expectations of students toward lecturers before receiving feedback (43). Happy feelings among students can lead to good self-reflection techniques and subsequently be able to improve communication skills (40, 47, 53, 54). Therefore, the type of non-verbal communication a lecturer uses can help students receive feedback on their performance in a calm and comfortable situation.

#### 4.2.3 Student’s feelings after receiving feedback

Students’ reflective writing also described how they felt after receiving feedback. After their lecturers provide feedback on their performance in communication skills, they continue to self-reflect on how to improve their skills (53). They appreciate feedback that has been delivered with a good and positive type of non-verbal communication. This is in line with previous studies that share similar findings on the appreciation shown by students to their teachers/lecturers/mentors, it turns out that they have received feedback well (41, 55–57).

Medical students in this study are also seen to have their perception of the importance of communication skills in the field of medicine. This may be due to the student’s experience in communicating with various parties, especially in preparing for an activity (23, 58, 59). Students who write about the importance of communication have high reflection skills and can be used as intrinsic motivation that allows the improvement process to be more successful (8, 51, 60). Therefore, if a student has a perception of the importance of something, it will facilitate a lifelong learning process and they will become medical students who constantly improve themselves.

Students also wrote about their determination to improve their weaknesses in communication skills. Students’ determination to improve their weaknesses is also associated with statements to improve their performance (9, 46). This is related to receiving good feedback from lecturers and the perception of the importance of communicating (61). Students also associate receiving good feedback from their lecturer through good non-verbal communication also increases their determination (36, 56). This is also in line with previous studies that show the good effects of NVC, which include creating a student who is aware of his own weaknesses and can work on being better (1, 50, 62–64). Therefore, NVC is very important to set a conducive learning environment for students’ self-development.

Improvements that have been written by students are based on self-reflection. The process of self-reflection usually fulfils three main conditions namely the nature of openness in seeing things from various angles, observing the performance of oneself and others and making good comparisons, and taking an objective approach in answering any questions or problems (40). It also needs to be accompanied by an organized plan to ensure the improvement process runs smoothly. This includes providing learning plans, implementation of strategies, plans, conducive learning spaces, repeated reflection processes, and continuous evaluation (65). Observations from this study are in line with previous studies that show that students are determined to improve their weaknesses and it is driven by a high level of reflection (66). Therefore, with detailed planning and support from lecturers, the improvement process planned by students will successfully improve their communication skills.

#### 4.2.4 Observation of types of non-verbal communication among lecturers

Students’ reflective writing was also found to have observed some types of non-verbal communication (NVC) of lecturers. This includes facial expressions, voice intonation, eye contact and smiles. This is in line with previous studies that show the types of NVC that are often observed by students (67). If compared with data from observations through a synchronous online platform, lecturers mostly exhibit facing the camera, moving hands while giving feedback and episodes pausing and looking left and right when remembering information. However, students only look toward cheerful facial expressions, which are accompanied by soft and calm voice intonation, eye gaze and a smile which provides a calm and appropriate atmosphere for feedback giving. This is in line with the findings of previous studies that show students only see facial expressions including the way of speaking, intonation and smile (5, 8, 62). Therefore, it is important for lecturers to always maintain a cheerful facial expression accompanied by a positive aura when giving feedback to students, whether through synchronous online platforms, asynchronous, video recordings or face-to-face methods.

A recent investigation conducted in Malaysia revealed a notable prevalence of non-verbal communication (NVC) within the context of Arabic language instruction as perceived by students. This observation underscores the significance of visual cues alongside auditory comprehension in face-to-face communication settings, wherein students not only rely on listening skills to grasp verbal information but also on the observation of NVC cues employed by their instructors (68). Similarly, a separate study focusing on the communication skills of medical students found that a considerable portion, ranging from 2 to 13%, required active support and encouragement from faculty members (69). This underscores the notion that effective communication skills are not inherently acquired, warranting medical schools to prioritize the cultivation of comprehensive communication abilities among future physicians. The various forms of non-verbal communication demonstrated by educators hold significance as they can influence students’ inherent tendencies to address deficiencies in communication. Reflecting on a cohort of medical students from the Faculty of Medicine at the National University of Malaysia who participated in a virtual patient software program, DxR Clinician, during their psychiatry rotation, the significance of interactions with instructors during debriefing sessions became apparent (70). While the group-based learning facilitated by the virtual patient software offered prompt feedback through assessment outcomes, students expressed a desire for additional opportunities for direct inquiry and personalized feedback from their instructors. Consequently, in online feedback sessions, student receptivity assumes significance, as it enables them to discern the congruence between verbal feedback and the instructor’s non-verbal communication cues.

#### 4.2.5 Application of non-verbal communication in the advancement of technology

In the near future, chatbots and other digital aid tools like artificial intelligence (AI) in medicine will be used extensively for doctor-patient communication. Majority of studies on the application of extended reality technology concentrate on its value in medical education; nevertheless, there is still much to learn about the genuine consequences of this technology’s use in face-to-face physician-patient interactions (71). A recent German study investigated on the attitudes of medical students toward AI and chatbots in medicine. The students thought that using chatbots as interactive diaries or therapy tools would be a useful way to lessen patients’ feelings of guilt, shyness, and anonymity while sharing sensitive and painful information. However, it was believed that interpersonal information would be lost and nonverbal contact with a chatbot could worsen doctor-patient relationships (72). Therefore, it was thought that the initial face-to-face interaction between the physician and patient was essential. It is also shown that artificial empathy can identify emotions in profile and 3/4 view photos, making it a potentially useful assessment tool for examining patient-doctor interactions in real-world clinical situations (73).

## 5 Limitations and recommendations

### 5.1 Findings of observation

All the lecturers involved in this study have known that they will be recorded while carrying out communication skills activities with students. Therefore, the type of NVC observed through online video recordings may seem contrived. However, there are two suggestions to overcome this problem. Observations that have been made by researchers only record the type of NVC that is made repeatedly over a long period. This is called repetitive behavior or habitual movement that is done unconsciously. For example, if a person touches the frame of the glasses 10 times in 5 min, that shows repetitive behavior and should be considered as a genuine finding.

The next suggestion that can be put into practice in future studies is to look at the types of NVC retrospectively. Virtual teaching and learning involved a lot of video recording for review purposes. Therefore, to obtain a holistic, unbiased and multi-angle observation, retrospective observation without the lecturer being aware that he is being recorded will add impact value to this study. This is because these studies generally show good types of NVC.

### 5.2 Increasing the number of samples

Since the observations in this study involved only 10 lecturers, if improvement can be made to the study sample, it can show a comparison of the types of NVC exhibited by younger lecturers and more senior lecturers. Increasing the number of samples can also diversify types of NVC between the sexes. This study also only looks at preclinical lecturers in communication skills activities. This can be expanded to observe the types of NVC among clinical lecturers and the acceptance of students who have more experience receiving good or bad feedback can also be discussed.

### 5.3 Focused discussion groups

The findings of this study were able to open the paradigm of future studies by forming focused discussion groups among medical students to see which types of NVC are more dominant and preferred, and how students receive feedback through various NVC shown by lecturers. The discussion group can also be focused on the lecturers to observe and find out why certain NVC is performed. This is to better understand whether the NVC exhibited can improve on how a message is interpreted to influence the intrinsic nature of students. This suggestion is expected to train lecturers to provide feedback well to students as a continuous assessment effort.

## 6 Conclusion

The study of observing the types of NVC online is indeed very important to explore the dominant types of NVC that can be identified by students to receive good feedback. Lecturers need to provide a calm and cheerful atmosphere to encourage students’ openness in receiving feedback and to make them feel comfortable. Positive feedback will give students a happy feeling. Facial expressions, voice intonation, frequent hand movements, eye contact and smiles are findings that are often observed among lecturers during feedback sessions. Students’ acceptance of these types of NVC were extracted from their reflective writing.

Reflective writing shows that students feel very anxious and nervous before receiving feedback because they think about their poor performance. This may be due to high expectations of oneself and fear of receiving negative evaluations. However, this increases their determination to improve their weaknesses. Since students can observe positive NVC from their lecturer (smile, soft voice intonation) which provides a calm atmosphere, they feel relieved and can accept the feedback that has been given. The importance of providing a calm and comfortable atmosphere will affect how students receive feedback. Therefore, this reflective writing complements the observation through a synchronous online platform that shows positive NVC observation from the lecturer. All the findings of this study are important to guide lecturers to convey information accompanied by good NVC.

## Data availability statement

The original contributions presented in the study are included in the article/Supplementary material, further inquiries can be directed to the corresponding author/s.

## Ethics statement

The studies involving humans were approved by Research Ethics Committee Universiti Kebangsaan Malaysia. JEP-2020-718. The studies were conducted in accordance with the local legislation and institutional requirements. The participants provided their written informed consent to participate in this study.

## Author contributions

NI: Conceptualization, Data curation, Formal analysis, Investigation, Methodology, Software, Validation, Visualization, Writing – original draft, Writing – review & editing. NM: Writing – original draft, Writing – review & editing, Data curation, Visualization. SB: Methodology, Supervision, Validation, Writing – original draft, Writing – review & editing. MB: Conceptualization, Formal analysis, Funding acquisition, Investigation, Project administration, Supervision, Validation, Writing – original draft, Writing – review & editing.
