# Supplementary material for: Beyond words: analyzing non-verbal communication techniques in a medical communication skills course via synchronous online platform
Source: Front Med (Lausanne). 2024 Apr 18;11:1375982. doi: 10.3389/fmed.2024.1375982 (PMC11064655; doi:10.3389/fmed.2024.1375982)
Supplement: Supplementary file 1 [file Data_Sheet_1.docx]

Supplementary Material

Beyond Words: Analyzing Non-Verbal Communication Techniques in a Medical Communication Skills Course via Synchronous Online Platform

Noor Akmal Shareela Ismail*, Nanthini Mageswaran, Siti Mariam Bujang, Mohd Nasri Awang Besar

*** Correspondence:** Noor Akmal Shareela Ismail: nasimail@ukm.edu.my

# Supplementary Figures and Tables

## Supplementary Table

| **Types of Non Verbal Communication** | **Yes** | **No** | **Notes**  **(Duration/Comments)** |
| --- | --- | --- | --- |
| **Body Position** | | | |
| Leaning forwards |  |  |  |
| Sitting upright |  |  |  |
| Always focused |  |  |  |
| Bending over |  |  |  |
| Sitting tilted to the left/right |  |  |  |
| **Facial expression** | | | |
| Looking directly into the camera |  |  |  |
| Smiling |  |  |  |
| Frowning |  |  |  |
| Laughing |  |  |  |
| Raising eyebrows |  |  |  |
| **Voice Intonation** | | | |
| Monotonous |  |  |  |
| Raising voice |  |  |  |
| Unclear voice |  |  |  |
| Dynamic voice |  |  |  |
| **Movement** | | | |
| Hands touching face |  |  |  |
| Hands touching chin |  |  |  |
| Hands touching nose |  |  |  |
| Hands at the mouth |  |  |  |
| Nodding the head |  |  |  |
| Shaking the head |  |  |  |
| Body swaying to the left/right |  |  |  |
| Body swaying to the front/back |  |  |  |
| Free movement of hand |  |  |  |
| Hands on the hair |  |  |  |
| Scratching the body |  |  |  |
| Scratching the forehead |  |  |  |
| Squinting eyes |  |  |  |
| Rubbing eyes |  |  |  |
| Touching shoulders |  |  |  |
| Touching frame of glasses |  |  |  |
| Clapping hands |  |  |  |
| Scratching behind the ears |  |  |  |
| Signalling OK |  |  |  |
| **Eye contact** | | | |
| Looking to the right |  |  |  |
| Looking to the left |  |  |  |
| Looking above |  |  |  |
| Looking downwards |  |  |  |
| **Paralinguistics** | | | |
| Word fillers *(urmm/”okay”/ lah/ “so”/”that’s it”*) |  |  |  |
| Sound of being shocked |  |  |  |
| Long breath of relieve |  |  |  |
| Pause for a moment |  |  |  |
| Clearing throat |  |  |  |
| Speaking in a fast pace |  |  |  |
| Speaking in a slow pace |  |  |  |

**Supplementary Table 1.** Checklist on the types of non-verbal communication

| **Theme** | **Sub-theme** | **Statement** |
| --- | --- | --- |
| **Students feelings before receiving feedback** | **Nervousness** | *…though I must admit I would be a little nervous if they had a stern facial expression. (K1P8)*  *I was feeling very nervous as I was worried if Prof would like what my partner and I did. (K1P9)*  *I felt very nervous and worried my performance (K1P10)*  *I felt very nervous and worries about the feedback of facilitator and my other friends on my task (K2P1)*  *I felt nervous because we thought this video and presentation was contributing marks for our evaluation (K2P2)*  *I was worried and nervous because I thought our acting in the video was quite awkward since we did it via Zoom but not face to face (K2P3).*  *I was very anxious whether it will be a positive comment or a negative one. (K2P8)* |
|  | **Restlessness** | *I was anxious and curious about how my teammates and our facilitator’s opinions on the task (K1P3)*  *I was already feeling anxious about what would be the feedback towards the outcome (K1P5)*  *I felt anxious and scared of what will my friends and my facilitators would (K1P6)*  *We all still feel anxious, scared, and worried when receiving feedback as we are worried about how the facilitator looks at us and perceives us (K2P4)*  *I was feeling very nervous thinking about how our facilitator, Dr Dain will be reacting and give feedbacks for my group and others (K2P8)* |
|  | **Fear** | *I was kind of scared as this was our first roleplay video (K2P2)*  *We all still feel anxious, scared, and worried when receiving feedback as we are worried about how the facilitator looks at us and perceives us (K2P4)* |
|  | **Apprehension** | *I was worried and nervous for this session because I scared that I would be the worst student for this task. (K1P1)*  *I was not confident whether my performance was good enough to be accepted. (K1P1)*  *I felt a little bad about myself as I could not give a proper presentation (K1P2)*  *Many things were running through my head thinking if there were parts that I could have done in better way, after looking at some of my peer’s performance (K1P9)*  *I think I’m not good at communication and need some improvement (K1P10)*  *I thought our acting in the video was quite awkward since we did it via Zoom but not face to face. (K2P3)* |
| **Students feelings when receiving feedback** | **Relief** | *I was able to receive the feedbacks without being triggered or feeling sad (K1P1)*  *I believe that it has helped me a lot and I still think the same way as I am right now (K1P3)*  *I would feel a slight relief whenever he/she puts on a smile on their face (K1P8)*  *A sense of satisfaction is how I feel after receiving feedbacks from my facilitator as I completed one of my assignments (K1P9)*  *I was grateful with his way of giving feedbacks as this made me feel relaxed throughout the session. (K2P3)*  *I was very glad to receive positive and motivating feedbacks from him regarding both of my group’s presentation. (K2P8)* |
|  | **Comfortable** | *Prof conveyed her feedbacks nicely to everyone. (K1P1)*  *I felt comfortable as our facilitator did not show any expression of displeasure on her face. (K1P2)*  *I am sure of is that my team members are now more comfortable to crack a joke among us (K1P3)*  *the facial expression and tone from the facilitator were really positive and full of motivation that makes me feel comfortable and able to receive the advice effectively. (K1P4)*  *That kind of eases us out of tension towards our feedbacks. (K1P5)*  *She sets the mood right. (K1P6)*  *I have never felt uneasy or disheartened by how my facilitators presented their feedbacks (K1P8)*  *This brought a great atmosphere for all of us during the session the other day. (K1P9)*  *I even felt very calm as looking Prof. smiled to us and tried to explain our mistakes. (K1P10)*  *to develop communication skill better and let us more comfortable to talk to each other online (K2P1)*  *I was grateful with his way of giving feedbacks as this made me feel relaxed throughout the session. (K2P3)*  *I was pleased that Dr. Dain responded positively to our work by giving feedbacks in such a comfortable way. (K2P3)* |
|  | **Happy** | *My group members and I feel very happy because receive a lot of positive feedbacks from our friends and facilitator (K2P1)*  *I am welcome and happy to receive any positive and negative feedbacks (K2P2)*  *His voice cue was not in an angry tone throughout the session. This made us feel more focused and excited throughout the session. (K2P8)* |
| **Students feelings after receiving feedback** | **Appreciation** | *I felt the feedback session was very meaningful and great (K1P2)*  *I believe that it has helped me a lot and I still think the same way as I am right now (K1P3)*  *Prof guided me towards the right path in enhancing my communication skills. (K1P9)*  *I am very happy and grateful to learn the ways of communication skills with my facilitator (K2P1)*  *Prof’s facial expression and voice cue motivate me to improve my communication skill and enable me to feel free to accept any negative feedback in the future because Prof always gave her feedbacks softly to everyone without interrupt our emotion. (K2P1)*  *He pointed out the parts we should improve such as eye contact, body language and the right way of approaching someone to start a conversation naturally. (K2P2)*  *Feedback from my facilitator was very valuable because it really helped to improve my communication skills (K2P8)*  *I strongly feel that having someone to guide us through and help us to improve ourselves is crucial for self-development (K2P10)* |
|  | **Perception** | *…which made me to understand my weakness and not feel embarrassed about it. (K1P2)*  *I believe that to receive a better feedback, it was supposed to be me as the one that needs a change (K1P3)*  *to know my weaknesses and strengths in communication skills to be more confident in improving my personal skills (K1P5)*  *open minded towards the acceptance of criticizing comments and viewing them from a humble angle (K1P7)*  *it is very important to communicate with eye contact and the appropriate speed which can make us deliver our message more clearly and easily during the conservation (K2P1)*  *we also learnt that we must adjust to different situations and audiences, especially later on when we start working as a doctor (K2P2)*  *I can see that the facilitator’s feedbacks motivate me to improve my communication skills (K2P3)*  *parts where we needed to improve such as body language and the right way of approaching people to start a conversation* |
|  | **Determination** | *To improve my performance from time to time (K1P4)*  *I think I’m not good at communication and need some improvement (K1P9)*  *I got to know that I must work on to improve my communication skills. (K2P2)*  *I recognise that I need to develop the confidence and learn the right way of communicating with others (K2P3)* |
|  | **Improvement** | *to hone those skills and practice them to achieve near perfection (K1P7)*  *I plan to continue improving my communication skills (K1P8)*  *I am sure now that this is something I am working on to be better. (K1P9)*  *I also believe that it is very important to accept ourselves learn from mistakes because avoid making the same mistake again in the future and continue to grow stronger as we through our life. (K1P10)*  *to take note form the negative feedbacks given and practice the knowledges that I learnt in my real life to improve my communication skills because practice makes perfect. (K2P1)*  *I will try to improve and sharpen my communication skills to become a better version of myself. (K2P2)*  *I felt that the feedbacks were beneficial for me to improve myself. (K2P4)*  *I will also try to make that the communication is short and straight to the point (K2P8)* |
|  | **Self-reflection** | *I am no longer afraid and nervous when receive feedbacks from someone (K2P1)*  *I realised and accepted our mistakes gracefully (K2P4)*  *I understand that I still have a long way to go to possess a strong and effective communication skills (K2P9)*  *I have learned a lot regarding communication skills and how to implement it in my daily life (K2P10)* |
| **Observation of lecturers’ types of non-verbal communication** | **Facial expression** | *I felt comfortable as our facilitator did not show any expression of displeasure on her face. (K1P2)*  *Prof’s facial expression was very calming, and I could feel the positive vibes from Prof (K1P1)*  *He always puts on a smiling face and passes his comments in a very polite manner as to not hurt our feelings (K2P3)* |
|  | **Voice intonation** | *…tone from the facilitator were positive and full of motivation that makes me feel comfortable and able to receive the advice effectively. (K1P1)*  *She delivers her comments in a calm facial expression and a gentle tone. (K1P5)*  *Soft spoken and encouraging towards our efforts and capabilities (K1P8)* |
|  | **Eye contact** | *One thing would be that making eye contact when appropriate is important when talking to someone to ensure that the conversation is bidirectional and for both to receive inputs from it. (K1P7)* |
|  | **Smile** | *Prof put on a pleasant smile, and I did not feel any sense of discomfort from her. (K1P5)* |

**Supplementary Table 2.** Thematic Checklist
